# Integrated care pathways: a new approach for integrated care systems

**DOI:** 10.3399/bjgp23X734925

**Published:** 2023-09-01

**Authors:** Christina van der Feltz-Cornelis, Emily Attree, Mel Heightman, Mark Gabbay, Gail Allsopp

**Affiliations:** Department of Health Sciences, Hull York Medical School, University of York, York; Institute of Health Informatics, University College London, London.; Ridgmount Practice and the Lawson Practice, London; Quality Improvement Clinical Lead, Central Camden Primary Care Network, PPI STIMULATE-ICP, London.; Consultant Respiratory Physician and Deputy Clinical Divisional Director for Medical Specialities, University College London Hospital, London.; Department of Primary Care and Mental Health, University of Liverpool, Liverpool; Academic Associate GP, Brownlow Health, Liverpool; National Institute of Health and Care Research Applied Research Collaboration North West Coast, Liverpool.; Royal College of General Practitioners, London.

One of the main aims of the newly formed integrated care systems (ICSs) is to improve population health and health care, establishing integrated care pathways (ICPs) for patients by devolving responsibility to local systems to ensure the care they provide is based upon their local populations’ health and care needs.^1^ The success of the ICSs in achieving this aim will depend on the collaboration of primary, community, and specialist care with community-based, voluntary partners and local authorities, who are responsible for public health and social care,^2^ and whether they take into account barriers to accessing care. The change towards local partners determining the patient pathways to improve outcomes will be significant. As a result, health and care pathways will depend on the ICS in which one lives. Much has been written about the aims and structure of the ICSs, and this is the latest in a long line of initiatives aiming to integrate care.^3^ Given the proposed autonomy for the local partners, one of the risks would be that ICSs would not change their approach to delivering ICPs, or make provision and demand-led decisions rather than based upon needs, evidence, or national guidance. To truly make a difference, the following aspects seem relevant.

## POPULATION DEMOGRAPHICS AND MULTIPLE CONDITIONS

ICPs would need to reflect the changing demographic of their local populations, taking age, gender, and social deprivation into account as they develop regionally, in addition to the local cultural mix and its influence on engagement with healthcare behaviours. Prioritisation of multimorbidity in the new ICSs is essential given the substantially increased incidence of multiple long-term conditions (LTCs) and in those accessing emergency care because of the rising age of the population. This has been exacerbated by the rise of new LTCs after COVID-19.^4^^,^^5^

## THE ROLE OF PRIMARY CARE

Primary care emphasises the role of the GP as the expert generalist in managing complex conditions. While much can be undertaken in the community, primary care will continue to need the support of specialist colleagues in a timely way to manage instability of progress, advise on new and specialist treatment options, and offer those diagnostic investigations, care pathways, and treatment options not available to GPs. ICPs should provide an overarching solution to managing multiple LTCs.

The bedrock of primary care is personalised, relationship-based care with care navigation, vertical and horizontal integration, and fluidity of specialist input.^6^^,^^7^ However, workload divisions, fractionation of tasks, and the pressure to focus on access and demand management have disrupted this essential continuity of care from GP teams. Furthermore, once a patient needs additional specialist support, they are then siloed into specialist teams in secondary care, having to navigate multiple teams and appointments with little coordination of that care, with associated delays and variable expectations of re-referral.

## CURRENT NHS CARE

As the NHS specialist care system is currently structured to support single diagnoses, clinics are redesigning the management of LTCs for the new ICS based on the principles of the Fuller report,^8^ aiming to integrate primary care within ICS structures while reducing health inequalities. For ICSs to succeed, the flow between primary care and specialty clinics should be supple, both vertically (including primary, community, and specialist care as required) and horizontally, to ensure a personalised approach to care where all systems and LTCs are considered by primary care and specialist teams working together.

For many LTCs, clinical networks with representation from primary, community, and secondary care services have served an important function in setting priorities and driving progress. Integrated care boards (ICBs) will need to ensure that these networks are adequately resourced and supported to encourage dissemination of best practice in place-based care, and mechanisms to allow networks to connect within and between regions will be important.

## DEALING WITH WAITING LISTS

There is traditionally a void between primary or community care on the one hand and specialist care on the other when specialist advice and treatment are needed. The backlog for diagnosis and treatment has risen even more since 2020 with an estimated number of over 6.73 million people currently waiting for treatment.^9^ To reduce the backlog, within NHS England, digital outpatient transformation, personalised patient-led follow-up, and the advice and guidance facility are promoted, designed to give rapid responses within 48 hours to GP queries.^10^ This quiet move away from shared-care decision making is concerning.^11^ The Royal College of General Practitioners position statement is clear that, if services such as advice and guidance are to be used, they must remain optional and be resourced appropriately for all clinicians involved in the pathway.^13^ As optional ‘add- on services’ as part of an integrated pathway, they could benefit patients and clinicians alike. Rather than looking at ‘quick fixes’ for the NHS, finding long-term solutions is more important than ever.

## LEARNING FROM POST-COVID CLINICS

During the pandemic, post-COVID clinics for the management of the long-term effects of COVID-19^14^ were set up.^15^ Post-COVID syndrome (long COVID) is a condition that can affect every system in the body and affects the whole person, including the ability to go to school, work, and look after themselves and their families.^16^ As support for this new disease, post-COVID clinics were set up from scratch using a novel approach focusing on the patient at the heart of the pathway, ensuring the wide-ranging clinical and rehabilitation teams fitted around the person, using a single point of access for care, unlike current practice for most LTCs. When well implemented, these services achieved highly integrated approaches. This integrated approach, which in some clinics involved primary, community, and secondary care clinicians coming together every week in a virtual multidisciplinary care meeting to discuss the patient needs, investigations, and collaborating on treatment plans, can point the way to redesigning care structures.^17^ Some services specifically funded GP leadership and others were GP led with input from specialists as appropriate.

## ASPECTS OF INTEGRATED CARE

Long COVID allows us to rethink our approach to the integrated care of patients with other LTCs that affect multiple systems and the whole person. Person-centred care with the patient at the heart of the care pathway, supported by a care coordinator, means patients do not have to chase multiple referrals themselves but instead receive diagnostic investigations and treatment for all related organ systems by a single pathway linking primary and secondary care together via a community trust. It would enable patients to maintain their primary relationship with their GP team, obtain the specialist investigations, treatment, and rehabilitation they need close to home, supported by a biopsychosocial model of care, putting the person at the heart of the process, especially if this pathway were based in the community. For example, psychiatric consultation models in primary care embedded in collaborative care were effective in comorbid depression and somatic LTCs.^18^ Additionally, they were particularly effective in case of medically not yet explained symptoms (MNYES) in primary care.^19^^,^^20^

Systems that have been trying to integrate this type of approach using specialist multidisciplinary team (MDT) clinics in primary care over the last few years include top-performing clinical commissioning groups (CCGs) and end-of-life care, as documented by the King’s Fund.^1^^,^^21^

If we applied the long COVID approach to all patients who require complex care, a hypothetical person with four LTCs, on the transfer of care to specialist services, would have a single care coordinator and attend a single clinic where all of their needs are met, with seamless transition between primary, community, secondary, and social care. Rather than the person navigating the system, care would be planned around the individual, with clinicians meeting to discuss the whole person and their needs in a coordinated approach to their care. This approach has been set up for the care of patients with long COVID.^17^ This has highlighted the value of clinically solid communities in setting priorities and driving progress in a disease area. It is essential for LTC clinical networks to tackle the needs of those with multimorbidity and multisystem disease. Now is the time to consider this approach for everyone with LTCs and complex needs, including MNYES.

## MODELS OF CARE PERSONALISED DEPENDING ON COMPLEXITY

Based upon the STIMULATE-ICP-Delphi study^12^ finding that patients want to feel heard, validated, and understood emotionally, relationship-based, whole-person care is at the heart of this. It requires clinician continuity to allow for trust to be built within repeated clinical/interpersonal interactions and to empower patients in self-care. Consultations that focus on shared understandings of needs, priorities, and management will continue to be at the core of good-quality integrated care so that the patient voice is not lost within multidisciplinary professional inputs.

We propose a new way to determine how integrated care should be delivered, as shown in Table 1, that could enable ICS colleagues to consider the personalisation of the patient care pathway.^12^ These six models range from limited to high complexity depending on the type of condition, the expected recovery time, how this is currently managed, and how we could consider changing current care to benefit the patient. Using these models would enable clinicians to begin to navigate a seamless, comprehensive approach to care, putting the person at the centre, thus ensuring the right level of care is delivered in the right place at the right time. Using this approach to a new disease such as long COVID could be the blueprint for an ICP for LTCs with different levels of complexity. See Box 1 for key messages.

**Table 1.** Potential models for ICPs managing recovery in long COVID and other LTCs^12^

**Example condition(s)**	**Expected recovery time**	**Gaps and suggestions for management**
**Model 1: Community**		
Community-acquired pneumonia (CAP)	Six months to fully recover in terms of fatigue	Primary care. There is currently no well-developed ICP, and there is a need to identify which cases require follow-up
**Model 2: Community-based multidisciplinary care**		
Acute insult such as post-myocardial infarction or significant musculoskeletal injury	Takes a medium course to resolution, between 1–2 years	Community multidisciplinary team providing physiotherapy with support for adjustment reactions, anxiety, and depressive symptoms if required
**Model 3: Community-based with integrated support**		
Chronic diseases, such as type 2 diabetes	With management, recovery can sometimes occur but it is rarely complete	Primary care by GP and diabetes nurse with complex cases supported in a specialist setting. There would be a need to identify such cases systematically within an existing ICP
**Model 4: Secondary care-supported community care**		
Chronic medical conditions such as rheumatoid arthritis or COPD	High disability, chronic course with exacerbations and relapses	Limited care provision, mostly in primary care, that needs hospital management in case of exacerbations, specialist medication, and later stages of care
**Model 5: Integrated care**		
Comorbid physical and mental disorders/LTCs, for example, uncontrolled diabetes and bipolar disorder	Chronic conditions with continuous high disability	There is an unmet clinical need for integrating psychiatric, psychological, and physical specialist teams: specialty mental health settings offer case-management for mental disorders but do not identify somatic illness related needs systematically; clinics for somatic treatment often have access to short-termpsychological treatments but not long-term ones; and critical workforce shortages of those with the required skillsets for patients with combined somatic and psychological problems exist. Current Improving Access to Psychological Therapies (IAPT) models of care need to be appropriately tailored for individuals with LTCs, and IAPT services are usually stand-alone and not integrated with other health services
**Model 6: Integrated care**		
Encompassing multimorbidity (that is, more than 2 LTCs), MNYES, or both.	High perceived disease burden	A multisystem specialist approach is needed in case of high disease burden requiring integrated physical and psychological healthcare provision. Currently, no consistent care pathway exists outside of primary care unless within post-COVID services. There is also a need to improve the delivery of anticipatory MDT care to avert serious outcomes in the most vulnerable

*COPD = chronic obstructive pulmonary disease. ICPs = integrated care pathways. LTCs = long-term conditions. MDT = multidisciplinary team. MNYEs = medically not yet explained symptoms*

**Box 1.** Key messages

The STIMULATE-ICP-Delphi study informs policymakers to help form integrated care systems (ICSs). Potential models for integrated care pathways (ICPs) managing recovery in long COVID and other long-term conditions are proposed. The proposed models envision moving from community level to a multisystem specialist approach, depending on condition, expected recovery time, and course.

It is clear that the role of public health in primary prevention, to be covered alongside secondary and tertiary prevention of LTCs, should not be ignored in the proposed model for integrated care. This should include public health expertise to explore data on prevalence, both expected and observed, to address potential health inequality issues. Public health services are nested in local authorities, but ICSs are starting to be expected to look at the big datasets for their area, to estimate expected versus known prevalence and risk factors among the population, to determine hotspots that would benefit from active engagement. In their early stages, such efforts would be best linked with rather than duplicating public health initiatives.

## A PARADIGM SHIFT IN INTEGRATED CARE SYSTEMS

A shift in current thinking needs to occur across the whole of the NHS, moving the integration of care vertically and horizontally across conditions depending on level of complexity and expected recovery time. For complex cases, integrated care clinical specialists supporting community settings, for example, specialist (respiratory, cardiology, renal, psychiatric) clinicians delivering integrated care in complex conditions and multimorbidity, should be available to community-managed patients without needing outpatient referrals, allowing for smooth exchanges between primary and specialist care. Moving care integration horizontally across all conditions, as suggested in model 6, outlined in Table 1, will enable us to realise the proper integration of care as we propose. Learning from a new disease, where we started with a blank canvas (as we did with long COVID) must enable us to think differently to improve care for everyone. Primary care could do much of this work, bringing the opinion of specialists, when needed, into the community, but only if funded and resourced appropriately and seen as equal and essential partners within the ICS at every level.

For this, efforts should not only be made to free up what is currently a limited supply of human resource and finance, but also consideration of how this would need to be balanced against other competing issues, costs, and workforce demands in secondary and primary care in enactment from a policy and funding perspective. Many long COVID services are GP led and fund GP post-COVID clinical leadership time specifically from post-COVID monies. While we are describing the scenario where well implemented, it is important to acknowledge the very variable implementation of services. Potential barriers to change in holistic care may need addressing, such as referral criteria, the role of primary care input, and leadership in long COVID clinics, and the variable implementation of services. Also, the size of ICPs compared with the required local pathways, national contracting of both primary and secondary care, and the influence of local political agendas on the ability to produce change would need addressing. This will be essential for the proposed models to be grounded in the reality of most clinicians’ daily lives.
